# Biomechanics of Red Blood Cells, Neutrophils, and Macrophages Under Oxidative Stress and Inflammation: A Narrative Review

**DOI:** 10.3390/biology15191710

**Published:** 2026-09-25

**Authors:** Viktoria Sergunova, Vera Shashkovskaya, Snezhanna Kandrashina, Mikhail Shvedov, Vladimir Inozemtsev, Ekaterina Sherstyukova

**Affiliations:** 1 Federal Research and Clinical Center of Intensive Care Medicine and Rehabilitology, V.A. Negovsky Research Institute of General Reanimatology, 107031 Moscow, Russia; verashashkovskaya@googlemail.com (V.S.); snezhanna.lyapunova@yandex.ru (S.K.); shvedovmike@bk.ru (M.S.); va.inozemcev@physics.msu.ru (V.I.); kmanchenko@yandex.ru (E.S.); 2 Department of Medical and Biological Physics, Sechenov First Moscow State Medical University, 119991 Moscow, Russia

**Keywords:** biomechanics, red blood cells, neutrophils, macrophages, oxidative stress, sepsis, cardiopulmonary bypass, IRF5

## Abstract

This narrative review summarizes current findings on the biomechanics of red blood cells (RBCs), neutrophils, and macrophages under oxidative stress and inflammation. Its cross-cell framework considers RBC injury, neutrophil activation, and macrophage remodeling as complementary components of a redox–inflammatory network linking microcirculatory alterations with innate immune responses. A focused, iterative search of PubMed, Scopus, Web of Science, and eLibrary.ru was used to identify relevant original and review articles. Oxidative and inflammatory stimuli remodel cell membranes and cytoskeletons and alter deformability, stiffness, adhesion, and migratory behavior. RBC adaptation to blood flow is closely linked to the maintenance of reversible deformability. Damaging factors, including prolonged storage, free iron, hemin, and oxidants, alter cell morphology, increase stiffness, and impair microcirculation. Hemolysis and the subsequent release of free iron and hemin further intensify oxidative damage to cell membranes. In neutrophils, priming, activation, and systemic inflammation induce changes in cell shape and physical properties, affecting capillary transit, tissue migration, and the propensity for NETosis. These alterations are associated with the severity of the inflammatory response. In macrophages, activation comprises a continuum of context-dependent functional states rather than two strictly separated M1 and M2 phenotypes. IRF5 regulates pro-inflammatory macrophage programs and represents a potential therapeutic target, although most evidence remains preclinical. The biomechanical parameters reviewed here should be considered experimental readouts or candidate biomarkers rather than clinically validated biomarkers. Their clinical translation requires method-specific standardization, independent reproducibility, and prospective validation.

## Simple Summary

To perform their functions, cells must change shape and resist mechanical forces. Changes in these properties can affect blood flow and immune system function. This review focuses on red blood cells, which carry oxygen, and two types of immune cells, neutrophils and macrophages. We examine how cell shape, flexibility, and surface properties change during inflammation and oxidative stress, a condition in which the cell’s defenses cannot adequately control excess reactive molecules. Changes in cell shape and flexibility may help cells perform their functions during inflammation, but they may also indicate damage. It remains unclear how reliably these properties can be used for diagnosis or to assess treatment outcomes. This highlights the need for consistent measurement methods and studies that track changes in cell properties in patients. Studying these properties may also help assess the quality of donated blood during storage.

## 1. Introduction

Oxidative stress and inflammation are major components of the pathogenesis of many critical conditions. RBCs and immune cells, including neutrophils and macrophages, undergo structural and functional changes in response to these processes. Such changes may impair RBC deformability as well as the migratory and protective functions of immune cells. The organization of the cell membranes and cytoskeleton is an important determinant of cellular functional and adaption to physicochemical stress.

This narrative review aims is to synthesize and interpret current evidence on the biomechanics of RBCs, neutrophils, and macrophages, and to describe the mechanisms of cellular damage under oxidative stress and inflammation.

RBCs, neutrophils, and macrophages were selected because they represent complementary components of the redox–inflammatory and microcirculatory response.

RBCs contribute to microvascular flow and oxygen transport, while their damage can lead to the release of hemoglobin, heme, and iron, therebly increasing oxidative and inflammatory stress [1,2]. Red blood cells contribute to microvascular flow and oxygen transport, while their damage can lead to the release of hemoglobin, heme, and iron, thereby increasing oxidative and inflammatory stress [3,4]. Neutrophils provide a rapid response to inflammatory stimuli and undergo activation-dependent changes in adhesion and migration [5,6], cell shape and deformability [7,8,9], and NET formation [7,10]. Macrophages clear damaged cells and contribute to the initiation, regulation, and resolution of inflammation [11,12,13,14]. Changes in their activation state are also associated with morphological and mechanical remodeling [15,16]. The main novelty of this review is the comparison of these three cell types within a shared biomechanical framework. We examine a common sequence consisting of oxidative or inflammatory stimulation, membrane and cytoskeletal remodeling, changes in cellular deformability, stiffness, adhesion, or morphology, and subsequent effects on microcirculation and immune function (Figure 1). Because most available studies examined these cell types separately, the proposed cross-cell relationships should be regarded as an integrative mechanistic model rather than a demonstrated causal pathway.

## 2. Literature Search Strategy

This article is a narrative review. To prepare this review, a structured literature search was conducted in July 2026 using PubMed, Scopus, Web of Science and eLibrary.ru. The search aimed to identify publications directly relevant to the topics addressed in the review rather than to provide exhaustive coverage of all records available in these databases. The final bibliography comprised 87 publications published between 1999 and 2026 and 1 methodological article. Given the narrative format of the review, its methodological quality was assessed using the Scale for the Assessment of Narrative Review Articles (SANRA) [17], an instrument specifically developed for evaluating narrative review articles. Searches were conducted in English and, for eLibrary.ru, in Russian. Different combinations of the following keywords were used: “erythrocytes”, “red blood cells”, “neutrophils”, “macrophages”, “biomechanics”, “deformability”, “stiffness”, “Young’s modulus”, “atomic force microscopy”, “scanning ion conductance microscopy”, “SICM”, “oxidative stress”, “inflammation”, “sepsis”, “hemolysis”, “hemin”, “transfusion”, “storage lesion”, “NETosis”, “macrophage polarization”, and “IRF5”. Publications were selected based on the relevance of their titles and abstracts to the aim of the review and their coverage of biomechanical, morphological, and functional changes in RBC or erythrocytes, neutrophils, and macrophages under conditions of oxidative stress, inflammation, sepsis, hemolysis, transfusion, and cardiopulmonary bypass. Publications outside the scope of the review and conference abstracts were excluded. The literature search and initial selection of publications were performed by two authors. Because the search was iterative and purposive and was not conducted as a systematic review, database outputs were not managed as a closed screening dataset, and the total numbers of retrieved and excluded records were not prospectively recorded. A flow diagram of the literature selection process is presented in Figure 2.

## 3. RBCs

### 3.1. RBC Membrane and Deformability

Red blood cells provide a convenient model for studying cell membrane damage and the associated changes in their biological properties, because their function in the circulation is largely determined by deformability and the integrity of the membrane–cytoskeleton complex. The red blood cell membrane is a two-dimensional structure comprising a lipid bilayer with integral proteins and an underlying cytoskeleton. The cytoskeletal network is formed predominantly by spectrin tetramers, actin, protein 4.1R, and linking proteins that attach to the membrane via band 3 with ankyrin involvement [18,19,20,21]. Weakening of the connection between the lipid bilayer and the cytoskeleton causes red blood cells to change shape and become less deformable. These cells lose part of their membrane and decrease in volume, thereby reducing deformability and making passage through capillaries more difficult [18,22,23] (Figure 3).

According to current concepts, red blood cell deformability is determined by a limited set of proteins that link the lipid bilayer to the cytoskeleton and participate in membrane organization and microvesiculation. This evidence is consistent with the concept of the membrane cytoskeleton as an integrated support system in which interactions between the lipid bilayer and cytoskeleton determine cell shape and deformability [24]. Although a red blood cell can experience considerable deformation during blood flow, the membrane itself is nearly inextensible: an increase in surface area of only 3–4% can already lead to hemolysis, highlighting the balance between high red blood cell deformability and preservation of membrane integrity [25,26]. Conversely, loss of membrane area through microvesicle budding decreases the surface-area-to-volume ratio, causes cell spherization, and reduces deformability; a similar mechanism of membrane loss is characteristic of hereditary spherocytosis (Minkowski–Chauffard disease) [24].

### 3.2. Mechanisms of RBC Adaptation and Membrane Remodeling During Physiological Aging, Long Storage, and Experimental Oxidative Injury

RBC remodeling is considered under three related but distinct conditions: physiological aging in the circulation, the multifactorial storage lesion developing ex vivo, and controlled chemical oxidative injury induced in vitro by tert-butyl hydroperoxide (tBuOOH). Ex vivo storage and tBuOOH exposure are not considered direct equivalents of inflammation in vivo. Rather, they model selected mechanisms, particularly oxidative modification of membrane components, remodeling of membrane–cytoskeleton interactions, increased stiffness, and impaired deformability [1,27], which may also contribute to erythrocyte dysfunction during systemic inflammation and sepsis [2,28,29]. The tBuOOH model provides reproducible, dose-controlled oxidative injury, but its strong, acute, and chemically defined action does not reproduce the variable intensity, spatial heterogeneity, temporal dynamics, and multicellular regulation of oxidative stress in vivo [27]. Under pathophysiological conditions, inflammatory mediators, activated leukocytes, endothelial interactions, altered shear stress, and endogenous antioxidant systems additionally influence RBCs [2,25,29]. Therefore, tBuOOH-induced changes should be interpreted as evidence of oxidative susceptibility rather than as a direct simulation of sepsis or other inflammatory diseases.

RBCs in the circulation continuously adapt to changing mechanical and metabolic conditions, preserving the ability to undergo reversible deformation while maintaining membrane strength [30,31]. During cell aging and damage, membrane changes occur, including Band 3 clustering, which promotes the recognition and clearance of RBCs by macrophages [32,33,34]. At the molecular level, oxidative modification of hemoglobin and membrane components can promote Band 3 phosphorylation and clustering, whereas lipid peroxidation disrupts lipid bilayer organization and alters membrane fluidity. These processes link oxidative stress to subsequent membrane–cytoskeleton remodeling; however, their detailed biochemical regulation is beyond the scope of this review, which focuses on measurable biomechanical, nanostructural, and morphological alterations and their functional consequences. During physiological aging, red blood cells gradually decrease in size, Na^+^/K^+^ ion pump function is impaired, and the cells become stiffer, increasing the probability of their removal from the circulation [32,35].

Similar changes are observed during prolonged storage of red blood cells. Most studies have shown that storage is accompanied by reduced cell deformability, with deterioration usually becoming apparent after two weeks of storage [1]. After transfusion, the stiffest and functionally impaired cells are removed more rapidly, whereas a substantial proportion of transfused red blood cells can recover their properties in the circulation, and the extent of recovery depends on the degree of initial damage [1,22].

Exposure to physicochemical factors such as oxidative stress, changes in ionic composition, and contact with artificial materials during ex vivo storage leads to remodeling of the lipid bilayer, protein interactions, and cytoskeletal organization. Contact with artificial materials alters cell morphology, membrane nanostructure, and stiffness, potentially reducing deformability and impairing blood microcirculation [36]. Reduced red blood cell deformability has also been reported in several pathological conditions, for example, sepsis [2,28,29].

The effect of oxidative stress can also be demonstrated in model experiments. In study [27], red blood cells were exposed to oxidative stress using tert-butyl hydroperoxide (tBuOOH). After incubation with tBuOOH, the ability of red blood cells to pass through microchannels designed to model narrow segments of the microvasculature was assessed. With moderate oxidant exposure, passage of red blood cells through the microchannels was essentially unchanged, whereas at higher levels of oxidation, the population separated into two groups: cells with a normal transit rate and cells that passed through the channels substantially more slowly. These “slow” cells increase the risk of microchannel occlusion [27].

A clinically oriented example is pathogen reduction of stored whole blood. In an in vitro study of partially leukoreduced low-titer group O whole blood treated with riboflavin and ultraviolet light, pathogen-reduced samples contained 63 ± 5% discocytes and 32 ± 6% echinocytes after seven days of storage, whereas hemolysis remained below 0.8% and Young’s modulus remained stable at approximately 5 ± 2 kPa [37]. Thus, the observed morphological remodeling was not accompanied by measurable stiffening of the cells.

These findings suggest that pathogen-reduced low-titer group O whole blood may be suitable for short-term storage and rapid transfusion during the prehospital and early hospital management of massive blood loss, where a balanced blood product with reduced infectious risk is required. Before routine clinical implementation, however, storage duration and standard quality indicators should be monitored, and prospective clinical studies are needed to confirm post-transfusion RBC recovery and patient outcomes.

From a biophysical perspective, red blood cell adaptation can be viewed as the maintenance of a balance between cellular deformability and membrane stability under continuously acting external mechanical forces. The ability of red blood cells to pass through capillaries without substantially increasing blood flow resistance is an important condition for normal function. Conversely, increased red blood cell stiffness may impair tissue perfusion and disrupt blood flow in microvessels and capillaries [1].

Classical biophysical models identify three principal determinants of red blood cell deformability. First, cell shape, particularly the surface-area-to-volume ratio. Second, cytoplasmic viscosity, which is determined by hemoglobin concentration. Third, the mechanical properties of the membrane itself, which depend on protein–lipid organization and membrane–cytoskeleton interactions [22]. Accordingly, reduced deformability may result from changes in cell shape, membrane modification, or cytosolic remodeling and is observed both during physiological aging and in pathological conditions; the mechanisms underlying increased stiffness during prolonged storage remain under active investigation [1].

The principal factors affecting erythrocyte membrane properties and deformability, together with the underlying mechanisms and functional consequences, are summarized in Table 1.

### 3.3. Free Iron and Hemin: Effects on RBCs

Studies of autologous transfusion in healthy volunteers showed that transfusion of RBCs stored for 40–42 days resulted in more pronounced signs of post-transfusion extravascular hemolysis than transfusion of RBCs stored for 3–7 days. This was accompanied by increased bilirubin and transferrin saturation and the transient appearance of non-transferrin-bound iron, whereas haptoglobin and lactate dehydrogenase changed only slightly [3,38,39]. Because non-transferrin-bound iron is redox-active, its accumulation may enhance reactive oxygen species generation and contribute to oxidative and infectious complications, particularly after repeated transfusions [3,40,41].

Hemolysis is an additional source of extracellular hemoglobin, heme, and iron. When physiological scavenging mechanisms are exceeded, these products accumulate and promote oxidative stress and inflammation. In sickle cell disease, extracellular heme acts as both a pro-oxidant and an RBC-derived damage-associated molecular pattern involved in vaso-occlusion and acute lung injury [4]. Related heme- and iron-mediated injury may also occur during cardiopulmonary bypass or prolonged mechanical circulatory support, in which hemolysis and non-lytic RBC damage are associated with impaired cellular mechanics, microcirculatory disturbances, and organ dysfunction [42].

Iron and hemin can oxidize membrane lipids and proteins, disrupt ion transport and cytoskeletal organization, and thereby reduce RBC deformability. Iron-induced morphological alterations have been demonstrated in RBCs from healthy donors [43,44,45,46], whereas intracellular iron overload in β-thalassemia minor has been associated with oxidative stress and abnormalities of Na^+^/K^+^-ATPase, Band 3, spectrin, protein 4.1, and actin [47]. Direct biomechanical evidence is derived mainly from controlled in vitro experiments. Exposure of healthy donor RBCs to Fe^2+^ (1700 μM) increased Young’s modulus by approximately 1.5-fold, whereas hemin increased it from approximately 10 ± 4 to 28.1 ± 7.5 kPa [43,48]. These findings support an association between iron- and hemin-induced oxidative injury and RBC stiffening; however, the magnitude of these effects depends on the experimental conditions and should not be directly extrapolated to in vivo concentrations.

### 3.4. Red Blood Cells During Cardiopulmonary Bypass

Cardiopulmonary bypass exposes RBCs to a combination of mechanical (high shear stress and turbulence), contact-related (interaction with artificial circuit materials), and biochemical (inflammation and complement activation) factors [49]. This produces a set of changes in cell shape, membrane nanostructure, and mechanical properties. Therefore, damage should be assessed using both rheological blood parameters and cell-specific parameters [42]. This was demonstrated in an AFM study in patients undergoing cardiopulmonary bypass (CPB) and CPB combined with hypothermic circulatory arrest [36]. After cardiopulmonary bypass, the apparent Young’s modulus of RBCs increased, with the most pronounced increase after normothermic CPB, whereas changes were less marked after CPB with hypothermic circulatory arrest. However, the proportion of discocytes was lower in patients undergoing CPB with hypothermic circulatory arrest. These findings indicate that morphological and mechanical parameters may reflect different aspects of RBC injury during cardiac surgery.

## 4. Neutrophils

### 4.1. Neutrophils—Rapid-Response Cells: Morphology, Activation, Adhesion

Neutrophils are heterogeneous innate immune cells whose lifespan and activation state depend on the physiological and pathological context [5,50,51,52,53]. Their recruitment requires rapid remodeling of the membrane and cytoskeleton. Under shear stress, rolling neutrophils form membrane tethers and slings that stabilize endothelial contact, while their deformability enables passage through narrow capillaries, particularly in the pulmonary circulation [5,6]. These processes provide a biomechanical basis for changes in cell shape, adhesion, and microvascular retention during inflammation.

This remodeling is also evident during neutrophil activation. The cells progress from an intact spherical state through successive stages of spreading and nuclear and membrane reorganization and, under strong or prolonged stimulation, may undergo NETosis. Although the general sequence of morphological changes is similar for different activators, its kinetics depend on the stimulus [7].

### 4.2. Changes in the Mechanical and Membrane Properties of Neutrophils in Response to Activating Stimuli

During priming and activation, neutrophils change not only their set of molecular markers but also their morphological and mechanical properties, including shape, size, stiffness, and deformability.

After priming and subsequent activation, neutrophils may exhibit biphasic mechanical changes. A transient phase of reduced cell size and increased compaction is followed by cell enlargement [10] and reduced stiffness. Under certain conditions, the cells may partially or completely recover their baseline biomechanical properties [8]. In the context of phagocytosis, however, the mechanical response differs. During particle uptake, neutrophils often become stiffer, and the increase in stiffness may begin before cell spreading is complete [9].

NETosis is accompanied by a distinct sequence of physical and mechanical changes. An in vitro study of primary human neutrophils from healthy donors, stimulated predominantly with PMA, showed that histone modification reduces the forces maintaining chromatin compaction, allowing chromatin to decondense and swell. The nuclear envelope then ruptures, and the expanding chromatin fills the cell, causing cell retraction and rounding. AFM measurements showed that the swelling chromatin generated an outward pressure of approximately 100–200 Pa, which increased until plasma membrane rupture. In parallel, progressive cytoskeletal disassembly was accompanied by a decrease in Young’s modulus from >1.5 to <0.3 kPa and a reduction in membrane tension from >0.35 to <0.07 mN m^−1^. Thus, the cell becomes mechanically softer while chromatin-generated pressure increases, and the combination of these processes facilitates plasma membrane rupture and NET release [54].

Oxidative stress is linked to neutrophil biomechanics through activation-dependent redox signaling and cytoskeletal remodeling. Some stimuli, such as PMA, induce NADPH oxidase-dependent ROS production, whereas calcium ionophores can induce NETosis through a largely NADPH oxidase-independent pathway; nevertheless, both responses involve reorganization of F-actin, the plasma membrane, and cell shape [5,7,10]. These structural changes occur together with stimulus- and time-dependent alterations in cell size, stiffness, and deformability [8,9]. Although the available studies do not establish a universal causal pathway from ROS generation to a specific mechanical phenotype, the accompanying biomechanical changes are functionally important because they influence neutrophil transit through the microcirculation.

These changes are directly relevant to microcirculation because neutrophils must deform readily to pass through capillaries approximately 5 μm in diameter or smaller. In the lungs, where the capillary network is particularly narrow, primed neutrophils with increased stiffness may be retained longer in the pulmonary capillary network. This prolonged retention is relevant to acute respiratory distress syndrome but is often reversible.

Over time, the cells become more deformable and begin to pass through capillaries into the interstitial space again. Thus, a reduction in stiffness is more important than a small increase in cell size [8].

A similar relationship between reduced neutrophil deformability and impaired microvascular transit has been observed in chronic metabolic disease. In patients with type 2 diabetes, unstimulated neutrophils exhibit reduced deformability, as indicated by prolonged transit through 8 μm pores. These alterations may contribute to microvascular dysfunction [55,56,57].

In children with type 1 diabetes, the proportion of activated neutrophils increases and the deformability of neutrophils in the resting state decreases. These changes decrease after the initiation of insulin therapy. The higher the glycated hemoglobin level, the lower the neutrophil deformability [58].

### 4.3. Neutrophils in Sepsis

Sepsis is characterized by systemic inflammation and dysregulation of the immune response; therefore, neutrophils are both markers and participants in the inflammatory process. Assessment of their biophysical parameters may be useful for understanding the transition from adaptive neutrophil activation to dysfunction.

In sepsis, neutrophils are exposed to high levels of circulating inflammatory mediators, leading to systemic priming. Ex vivo experiments have shown that plasma from patients with bacterial sepsis induces NET release by neutrophils from healthy donors. This effect is strongest after exposure to plasma from patients who subsequently died from sepsis and is associated with more severe organ dysfunction assessed using the SOFA score. In addition, these patients show shifts in neutrophil phenotype, including reduced expression of CD14, CD16, and CD64, indicating systemic remodeling of the immune response characteristic of severe sepsis [59]. Indices calculated from neutrophil, lymphocyte, and platelet counts may be associated with sepsis severity but represent indirect hematological markers rather than measurements of neutrophil biomechanics [60,61].

The term N1-like and N2-like are used here only as a simplified, context-dependent descriptors of neutrophil functional states and not as a biomechanical classification, because the cited studies primarily report inflammatory markers and functional phenotypes rather than direct measurements of neutrophil mechanics [62,63,64,65,66,67,68,69].

Additional evidence linking sepsis severity with neutrophil morphology has been obtained in neonatology. Atomic force and fluorescence microscopy showed that plasma from infants with generalized infection increases the tendency of neutrophils to undergo NETosis, accompanied by increased nuclear area and pronounced changes in membrane roughness. In contrast, plasma from patients with localized foci of infection has a more stabilizing effect on the cells. Such microscopy parameters were noted to be potentially useful for assessing the severity of the septic process [70].

Accordingly, measurements of neutrophil stiffness, deformability, and pore transit are considered direct biomechanical evidence, whereas surface roughness, nuclear area, NET formation, phenotypic markers, and blood cell count-derived indices are interpreted separately and do not directly characterize cellular mechanics.

Overall, these findings indicate that neutrophil activation and disease-associated stimuli are accompanied by interconnected changes in cellular mechanics, membrane properties, morphology, and functional state. The principal alterations observed during activation and under pathological conditions are summarized in Table 2.

Direct biomechanical changes during neutrophil activation, priming, and phagocytosis were mainly examined in primary neutrophils isolated from healthy donors and stimulated under controlled in vitro conditions [7,8,9,10]. Studies in diabetes evaluated deformability directly in neutrophils obtained from patients [57,58]. By contrast, the sepsis studies in which healthy donor neutrophils were exposed to patient plasma represent ex vivo plasma-transfer models [59,70].

## 5. Macrophages

### 5.1. Macrophage Plasticity, Polarization, and Biomechanical Remodeling

Macrophages are heterogeneous cells whose functional characteristics are determined by their developmental origin, tissue microenvironment, metabolic state, activating signals, and duration of stimulation [11,12,13,14]. Accordingly, macrophage activation is better represented as a multidimensional continuum of functional states than as a linear transition between two fixed M1-like and M2-like states [15]. Individual macrophages may simultaneously express inflammatory, regulatory, phagocytic, and tissue-repair programs, particularly under complex in vivo conditions. The M1/M2 paradigm is therefore used here only as a simplified framework for describing predominant response patterns.

In commonly used experimental models, LPS or interferon-γ induce M1-like states characterized by the production of pro-inflammatory mediators, including TNF-α, IL-1β, and IL-6, and increased expression of STAT1 and nitric oxide synthase 2 [11,14,71]. Prolonged predominance of these programs may contribute to chronic inflammation and tissue injury. By contrast, stimulation with IL-4, IL-10, or TGF-β is used to generate different M2-like states associated with the limitation of inflammation, extracellular matrix remodeling, and tissue repair [11,14]. The conventional M2 category has been further divided into M2a, M2b, M2c, and M2d subtypes; however, these labels describe experimentally induced response patterns rather than discrete macrophage populations in vivo. Excessive activation of reparative programs may also promote pathological tissue remodeling through the production of chemokines, matrix metalloproteinases, fibronectin, and collagen [72]. Macrophage activation programs are accompanied by remodeling of the actin cytoskeleton and cell–substrate interactions. These changes influence cell spreading, elongation, adhesion, migration, and phagocytosis and may be reflected in morphology, surface characteristics, adhesion, and the apparent Young’s modulus measured by AFM [11,15,16]. Redox-sensitive signaling pathways may contribute to this remodeling during inflammation. However, direct evidence that oxidative stress itself causes specific changes in macrophage Young’s modulus remains limited. Moreover, biophysical properties do not independently define a particular macrophage state. Similar mechanical characteristics may occur in cells with different molecular programs, whereas cells assigned to the same M1-like or M2-like category may respond differently depending on their origin, substrate, culture conditions, and stimulation protocol. These model-dependent differences are illustrated by AFM studies. In RAW 264.7 cells, unstimulated M0 macrophages showed a relatively low Young’s modulus, whereas experimentally induced M1-like cells showed a higher modulus and a more rounded and flattened morphology. M2-like cells were more elongated and showed a smaller increase in Young’s modulus and adhesion [16]. In primary murine bone marrow-derived macrophages, however, the Young’s modulus did not differ significantly between unstimulated M0 and LPS-induced M1-like cells. Macrophages induced toward an M2-like state with IL-4 and IL-10 had a higher Young’s modulus, greater length, and lower height than M0 and M1-like cells [15]. These contrasting findings indicate that there is no universal biomechanical hierarchy of M0, M1-like, and M2-like macrophages. Findings obtained using established cell lines should therefore be interpreted separately from those obtained using primary and tissue-resident macrophages. RAW 264.7 cells provide a reproducible model for controlled stimulation and biomechanical measurements, but prolonged culture and cell-line-specific characteristics may modify cytoskeletal organization and cellular responses. Primary murine bone marrow-derived macrophages represent a distinct experimental model but do not reproduce the ontogeny and organ-specific microenvironment of tissue-resident macrophages. Tissue macrophages are additionally influenced by the extracellular matrix, neighboring cells, and combinations of local signals [11]. The patient-derived studies discussed in this review primarily evaluated macrophage markers, inflammatory mediators, or IRF5 expression rather than direct mechanical properties [73,74]. Consequently, biomechanical findings obtained using RAW 264.7 cells or murine bone marrow-derived macrophages cannot be considered established characteristics of human tissue macrophages. Overall, macrophage activation programs are associated with coordinated changes in cytoskeletal organization, morphology, adhesion, and mechanical properties. However, these relationships are context- and model-dependent and cannot be reduced to fixed biomechanical signatures of M1-like and M2-like states.

### 5.2. Regulation of the Inflammatory Response and Macrophage Activation State: The Role of IRF5 and Its Relationship to Cellular Characteristics

Within the model of macrophage activation, IRF5 should be considered a regulator of pro-inflammatory transcriptional programs rather than a binary switch between M1-like and M2-like states. IRF5 inhibition may shift the molecular and functional profile of macrophages toward a less inflammatory state. However, changes in individual markers or mechanical properties do not by themselves demonstrate complete conversion from an M1-like state to an M2-like state.

IRF5 regulates Toll-like receptor-dependent pro-inflammatory transcriptional programs, particularly the expression of inflammatory cytokines, and may also contribute to type I interferon responses in a context-dependent manner [75,76,77]. Higher IRF5 expression has been reported in experimentally generated M1-like macrophages, macrophages from the synovial fluid of patients with osteoarthritis, and intestinal macrophages from neonates and mice with necrotizing enterocolitis [73,74]. siRNA-mediated IRF5 knockdown reduced pro-inflammatory markers in macrophage models [78], whereas genetic IRF5 deficiency or nanoparticle-delivered IRF5 siRNA reduced inflammation and tissue injury in preclinical mouse models [74,79,80].

In primary murine bone marrow-derived macrophages, siRNA-mediated IRF5 knockdown increased Young’s modulus in M0 and M1-like cells, but not in M2-like cells, and was accompanied by morphological remodeling and increased surface roughness [15]. These findings demonstrate an association between IRF5 modulation and biomechanical remodeling but do not show that increased stiffness causes a transition toward an M2-like state.

The translational interpretation of macrophage biomechanical data requires particular caution. Direct AFM measurements were obtained predominantly using the murine RAW 264.7 cell line and primary murine bone marrow-derived macrophages under controlled in vitro polarization conditions [15,16]. THP-1 cells provide an additional controlled human monocytes cell-line model [71], but they do not reproduce the full phenotype of primary tissue macrophages. Evidence supporting IRF5 inhibition as a therapeutic approach is derived mainly from mouse models [74,79,80]. Patient-derived evidence primarily concerns IRF5 expression or macrophage phenotype in clinical tissue samples rather than direct measurements of macrophage mechanical properties [73,74]. Thus, current findings support a mechanistic association between macrophage regulatory programs and biomechanical remodeling, but their applicability to human tissue macrophages and disease severity requires further clinical validation.

Table 3 summarizes the functional, molecular, morphological, and biomechanical characteristics of M0, M1-like, and M2-like macrophages, together with the effects of IRF5 modulation.

## 6. Methodological Considerations in Comparing Cell Biomechanics

Cellular stiffness and deformability are related but not identical characteristics. Each biomechanical method applies a specific type of mechanical load over a particular spatial and temporal scale. Therefore, its result reflects a different combination of membrane properties, cytoskeletal organization, cytoplasmic viscosity, cell geometry, and cell-surface interactions [82]. Measurements obtained using different methods should consequently not be interpreted as direct equivalents.

Across the three cell types, oxidative stress is linked to membrane and cytoskeletal remodeling, but the strength of evidence differs: this relationship is most directly characterized in erythrocytes, whereas in neutrophils and macrophages it is more stimulus-dependent and is often inferred from parallel changes in redox signaling and cellular mechanics [7,8,9,10,15,16,27,48,83].

Atomic force microscopy measures the local mechanical response of a cell to indentation. Force–indentation curves can be used to calculate an apparent Young’s modulus, but the resulting value depends on the contact model, probe shape and size, indentation depth, loading rate, measurement position, substrate, and sample preparation [48,82,84]. AFM provides high spatial resolution and can identify mechanical heterogeneity within and between individual cells. However, its throughput is relatively low, cells usually require immobilization, and local indentation does not directly reproduce whole-cell deformation during passage through a capillary or microchannel.

Scanning ion conductance microscopy (SICM) uses an electrolyte-filled nanopipette and ion-current feedback to maintain a defined distance between the probe and the cell surface. Conventional SICM primarily provides non-contact topographic imaging of living cells in physiological liquid conditions. In pressure-based nanomechanical modes, controlled hydrodynamic pressure generated through the nanopipette produces local cell deformation, from which compliance, viscoelastic properties, or an apparent Young’s modulus can be estimated [85]. This approach reduces direct probe-sample contact and is therefore suitable for repeated measurements of soft or fragile living cells. However, SICM is generally a low-throughput, single-cell technique. Its quantitative results depend on nanopipette geometry, applied pressure, electrolyte composition, cell immobilization, substrate effects, spatial resolution, and the mechanical model used for data interpretation. Furthermore, standard topographic SICM does not itself provide a direct measure of stiffness. SICM-derived local mechanical parameters should therefore not be considered interchangeable with whole-cell deformability measured by ektacytometry, filtration, microfluidics, or deformability cytometry.

Ektacytometry evaluates the elongation of an erythrocyte population under defined shear stress, usually by measuring a dimensionless elongation index. This index depends on membrane mechanics but is also influenced by the surface-area-to-volume ratio, intracellular viscosity, osmolality, temperature, and viscosity of the suspending medium [2,86]. Because conventional ektacytometry provides a population-averaged signal, small subpopulations of poorly deformable erythrocytes may remain undetected. Moreover, the elongation index is not equivalent to Young’s modulus, and conventional ektacytometry is not generally applicable to nucleated immune cells.

Microfluidic methods assess cell deformation and passage through channels, constrictions, or artificial microvascular networks. Their outputs may include transit time, velocity, deformation index, critical pressure, occlusion frequency, or network perfusion rate. These measurements can reproduce aspects of microvascular confinement and reveal single-cell heterogeneity. However, the results depend on channel geometry, pressure or flow rate, medium viscosity, cell size and shape, adhesion to channel surfaces, and device material [2,83,86]. Consequently, measurements obtained using different microfluidic devices are not automatically comparable. Direct experimental comparisons have also shown that ektacytometry and filtration measurements do not necessarily predict perfusion through a microfluidic capillary network [87].

Filtration methods determine the ability of a cell suspension to pass through membranes with defined pores. Filtration time, flow rate, pressure, or the proportion of retained cells can be used as measures of filterability. These methods are relatively simple and can analyze large cell populations. However, the result combines deformability with cell size, shape, concentration, aggregation, adhesion, and progressive pore obstruction [2,86,87].

Deformability cytometry, including real-time deformability cytometry, measures the hydrodynamic deformation of individual suspended cells at high throughput. It usually provides cell deformation together with cell size, while an apparent elastic modulus may be derived using an appropriate mechanical model [8,82,88]. The result depends on strain rate, channel geometry, medium viscosity, image segmentation, cell size, and the assumptions of the mechanical model. Different deformability cytometry platforms can show different sensitivities to the same cytoskeletal alteration because they expose cells to different loading conditions and deformation time scales [85].

Table 4 summarizes representative quantitative findings together with the methods, cell models, and experimental conditions. The values are presented separately for each study because differences in measurement conditions limit direct numerical comparison.

## 7. Limitations

Direct quantitative comparisons between studies remain limited because biomechanical measurements are sensitive to both pre-analytical and analytical conditions. The choice of anticoagulant, time from sampling, temperature, cell isolation and activation procedures, substrate and medium properties, probe geometry and loading rate, shear stress or channel geometry, instrument settings, and data-analysis models may influence the reported values [2,8,16,48]. Consequently, study-specific measurements should not be combined or interpreted as common clinical thresholds without methodological harmonization. Interlaboratory reproducibility studies are required before reference ranges or clinically applicable cutoffs can be established.

## 8. Conclusions

Thus, the evidence summarized in this review suggests that alterations in the biomechanical and membrane properties of erythrocytes and immune cells may reflect both adaptive responses to injurious stimuli and pathological mechanisms contributing to microcirculatory and inflammatory dysfunction. Although erythrocytes, neutrophils, and macrophages perform distinct functions, membrane and cytoskeletal remodeling in all three cell types is closely associated with alterations in surface properties and functional state. At present, biomechanical parameters should be regarded primarily as experimental readouts rather than clinically validated biomarkers; their translational potential requires standardized measurements, longitudinal clinical studies, and integration with molecular and functional markers.

## Figures and Tables

**Figure 1:**
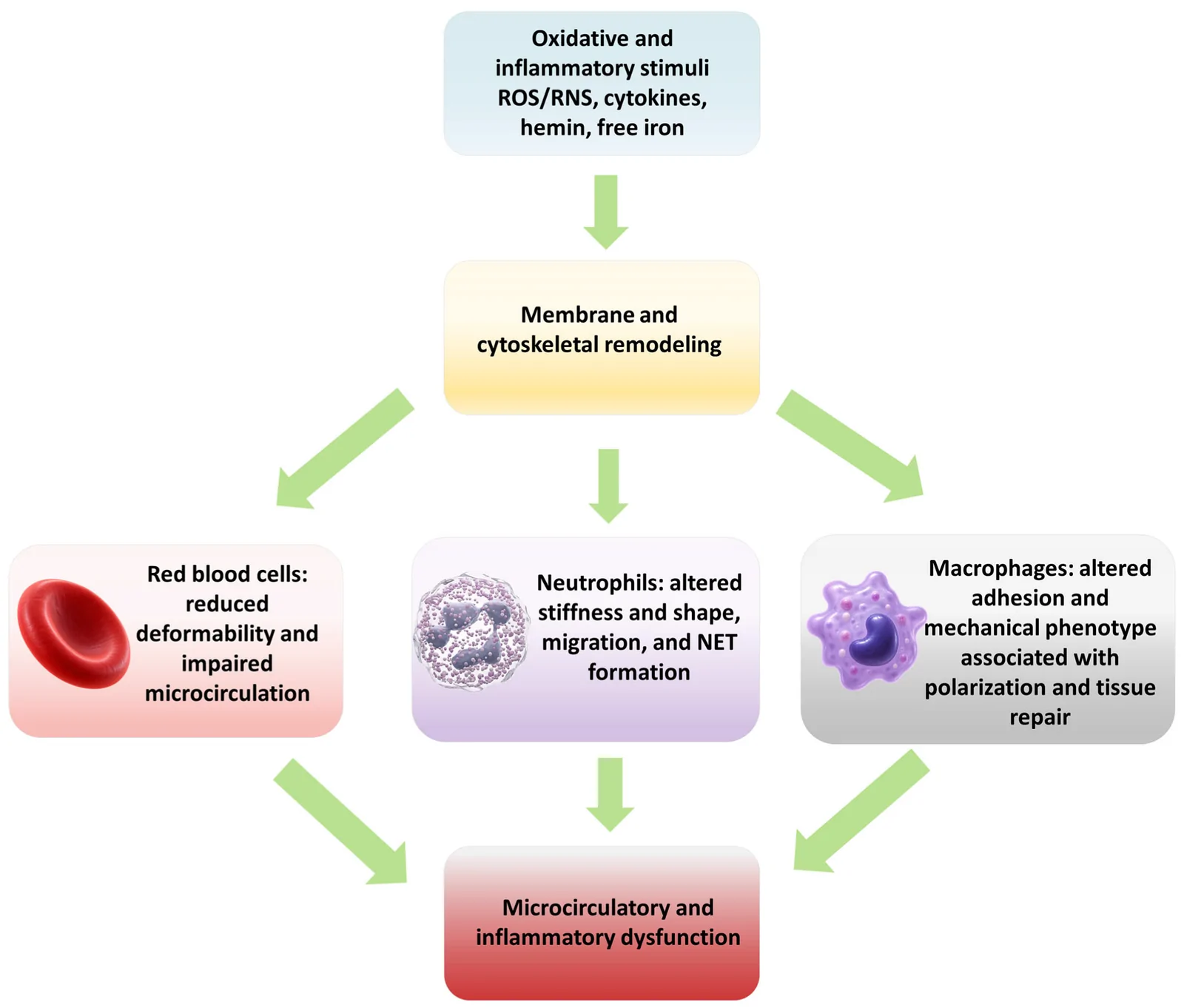
Common pathways linking oxidative and inflammatory stimuli to biomechanical remodeling of red blood cells, neutrophils, and macrophages.

**Figure 2:**
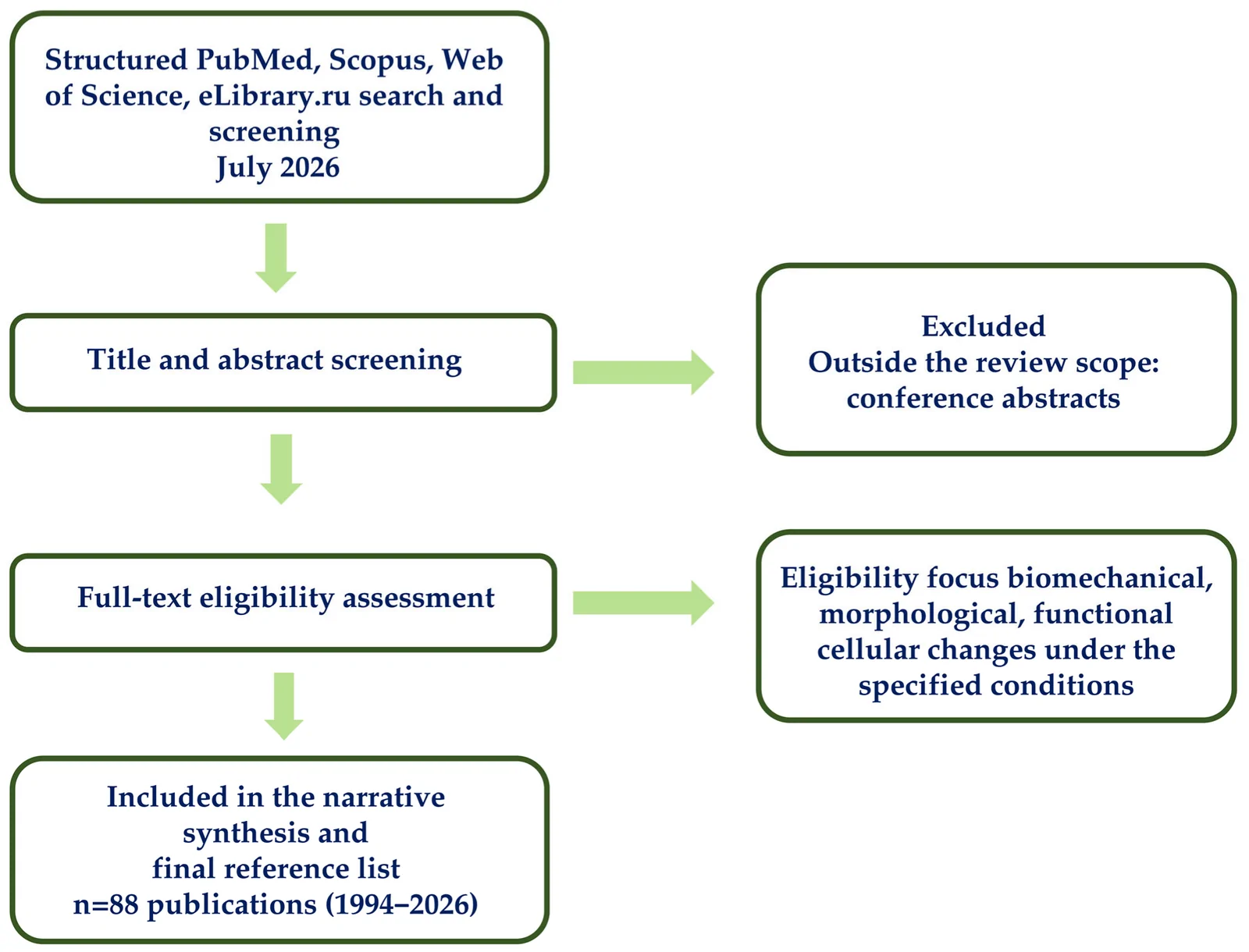
Block diagram of literature inclusion.

**Figure 3:**
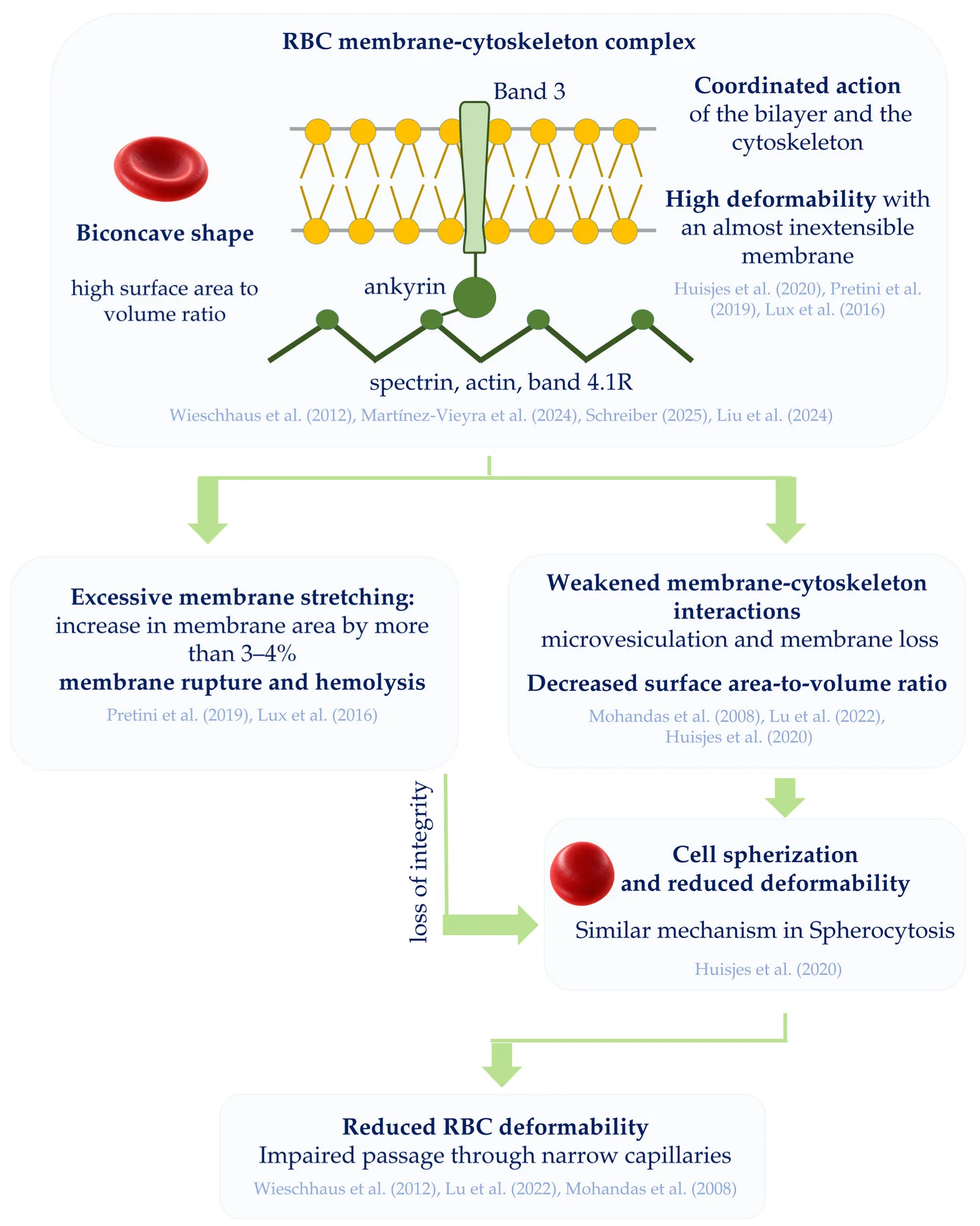
RBC membrane–cytoskeleton architecture and mechanisms underlying reduced deformability [18,19,20,21,22,23,24,25,26].

**Table 1:** Factors affecting RBC membrane properties and deformability.

Factor or Condition	Membrane and Cellular Changes	Changes in Biophysical Properties	Adaptive Response or Functional Consequence	Ref.
Physiological mechanical and metabolic stresses	Coordinated rearrangement of the membrane and cytoskeleton while maintaining the integrity of the membrane–cytoskeleton complex	Reversible deformation without a substantial loss of mechanical stability	Preservation of the ability to pass through narrow capillaries and maintain microcirculation	[26,30]
Physiological RBC aging	Band 3 clustering, reduction in cell size, and impaired Na^+^/K^+^ pump activity	Increased stiffness and reduced deformability	Recognition of altered erythrocytes by macrophages and their removal from the circulation	[31,32,33,34]
Prolonged storage	Accumulation of structural and metabolic membrane damage, the severity of which increases with storage duration	Reduced deformability, which generally becomes evident after two weeks of storge	Following transfusion, the most severely damaged cells are rapidly cleared, whereas some erythrocytes can partially recover their properties in the circulation	[21,35]
Contact with artificial materials	Changes in cell morphology, membrane nanostructure, protein–lipid interactions, and cytoskeletal organization	Increased stiffness and potentially reduced deformability	Impaired passage through microvessels and possible disruption of microcirculation	[1]
Sepsis and systemic inflammation	Alterations in membrane and cytoskeletal structures, the severity of which depends on the stage and severity of the pathological process	Reduced RBC deformability	Increased resistance to microvascular blood flow and potentially impaired tissue perfusion	[28,36]
Moderate oxidation induced by tBuOOH	Oxidative modification of cellular components without a marked impairment of passage through microchannels	No substantial change in the rate of erythrocyte passage through microchannels	Preservation of functional passage under conditions of compensable oxidative damage	[29]
Severe oxidation induced by tBuOOH	Heterogeneous damage within the erythrocyte population	Formation of two subpopulations: cells with normal and markedly reduced microchannel transit velocities	Slowly transiting cells may increase the likelihood of microchannel obstruction	[29]
Riboflavin/UV pathogen reduction followed by 7-day storage of low-titer group O whole blood	Decreased proportion of discocytes and increased proportion of echinocytes; hemolysis remained below 0.8%	Young’s modulus remained stable at approximately 5 ± 2 kPa	Morphological remodeling was not accompanied by measurable cell stiffening. The clinical relevance requires prospective validation	[37]
Altered cell geometry	Reduction in the surface-area-to-volume ratio resulting from changes in cell shape or membrane loss	Limited ability to undergo reversible deformation	Cell spherization and impaired passage through narrow capillaries	[21,35]
Altered cytosolic viscosity	Changes in the concentration and intracellular organization of hemoglobin	Increased internal resistance to deformation	Reduced ability of erythrocytes to adapt to the geometry of microvessels	[21]
Disruption of the protein–lipid organization of the membrane	Modification of membrane proteins, the lipid bilayer, and membrane–cytoskeleton interactions	Increased stiffness and reduced mechanical stability of the membrane	Disruption of the balance between erythrocyte deformability and membrane strength	[21,35]

**Table 2:** Changes in the mechanical, membrane, and functional properties of neutrophils during activation and in pathological conditions.

Exposure or Condition	Mechanical and Morphological Changes	Functional and Phenotypic Changes	Biological or Clinical Significance	Ref.
Priming and subsequent activation	Biphasic mechanical response: a transient reduction in cell size and cellular compaction is followed by an increase in cell size and a decrease in stiffness	Under certain conditions, the initial mechanical state may be partially or completely restored	The reversibility of these changes may allow neutrophils to retain their ability to pass through the microcirculation	[7,10]
Phagocytosis	Increased stiffness, which may begin before cell spreading is complete	The mechanical response differs from the decrease in stiffness observed under some other activation conditions	Increased stiffness may temporarily impair the deformability of activated cells	[8]
Neutrophil priming in the pulmonary microcirculation	Increased stiffness, a moderate increase in cell size, and prolonged retention in narrow pulmonary capillaries	Stiffness may subsequently decrease, thereby restoring capillary transit	This response may be relevant to acute respiratory distress syndrome; a reduction in stiffness appears to be more important for restoring capillary passage than changes in cell size	[10]
Type 2 diabetes	Reduced baseline neutrophil deformability and prolonged transit through 8 µm pores	Changes are observed without additional stimulation of the cells	These alterations may contribute to impaired microcirculation and the development of microvascular complications	[9,55]
Type 1 diabetes in children	Reduced deformability of resting neutrophils	Increased proportion of activated neutrophils and an inverse relationship between glycated hemoglobin levels and neutrophil deformability	The severity of these changes decreases after the initiation of insulin therapy, indicating that they are partially reversible	[56]
Plasma from neonates with generalized infection	Increased nuclear area and pronounced alterations in membrane surface roughness	Enhanced susceptibility of neutrophils to NET formation	Morphological and membrane parameters may reflect the severity of the septic process	[66]
Plasma from neonates with localized infection	Less pronounced changes in cell morphology and membrane roughness than those induced by plasma from neonates with generalized infection	A more stabilizing effect on neutrophils	Microscopy-derived parameters may facilitate differentiation between localized and generalized infection	[66]

**Table 3:** Model-specific functional and biomechanical characteristics of experimentally induced macrophage states and the effects of IRF5 modulation.

Macrophage State or Intervention	Inducing and Regulatory Signals	Molecular and Functional Characteristics	Morphological and Biomechanical Characteristics	Adaptive or Pathological Significance	Ref.
Unpolarized M0 macrophages	Absence of phenotype-specific polarizing stimuli; retention of the ability to respond to microenvironmental signals	Baseline functional state capable of transitioning toward different activation states	RAW 264.7 cells exhibit a relatively low Young’s modulus and high deformability. In primary bone marrow-derived macrophages, Young’s modulus does not differ significantly between the M0 and M1- like states	Provide a cellular reserve for adaptive polarization according to the nature of tissue injury or inflammatory stimuli	[70,72]
Pro-inflammatory M1-like state	Lipopolysaccharide and IFN-γ; involvement of STAT1-, IRF3-, and IRF5-dependent signaling pathways	Secretion of TNF-α, IL-1β, and IL-6; increased expression of STAT1 and NOS2; involvement in pathogen elimination and the development of Th1 responses	In RAW 264.7 cells, M1-like polarization is associated with an increased Young’s modulus and a more rounded and flattened morphology. In primary bone marrow-derived macrophages, Young’s modulus is similar between the M1-like and M0 states	Required for antimicrobial defense; prolonged predominance of the M1-like state may promote chronic inflammation and tissue injury	[12,13,15,70,72]
Anti-inflammatory and reparative M2-like state	IL-4, IL-10, and TGF-β, depending on the functional subtype	Secretion of IL-10 and TGF-β; involvement in the resolution of inflammation and tissue repair; subdivision into M2a, M2b, M2c, and M2d state	In RAW 264.7 cells, M2 polarization is associated with a slight increase in Young’s modulus and adhesion, as well as an elongated morphology. Primary M2-like macrophages exhibit a higher Young’s modulus, greater maximum cell length, and lower cell height than M0 and M1- like states	Promotes the resolution of inflammation and tissue repair; excessive M2-like polarization may contribute to pathological tissue remodeling and fibrosis	[12,14,70,71,72]
Dynamic spectrum of macrophage activation states	Changing combinations of cytokines, microbial products, extracellular matrix components, and other microenvironmental signals	Alterations in surface-marker profiles, secreted mediators, and intracellular signaling pathways	Cytoskeletal remodeling is accompanied by changes in cell shape, adhesion, height, length, and mechanical stiffness	Enables macrophages to adapt to successive phases of inflammation, antimicrobial defense, and tissue repair	[70,71,72]
IRF5-associated inflammatory programming and experimental inhibition	TLR-mediated IRF5 activation; experimental IRF5 inhibition, mainly using siRNA	IRF5 promotes pro-inflammatory transcriptional programs. Its inhibition shifts macrophages toward a less inflammatory profile	In primary murine macrophages studied in vitro, IRF5 silencing altered Young’s modulus, cytoskeletal organization, and membrane roughness. These associations do not establish that stiffness drives macrophage state transitions	The available therapeutic evidence is preclinical and is derived mainly from experimental mouse models	[15,74,75,76,77,78,79,80,81]

Notes: The M0, M1-like, and M2-like designations refer to experimentally defined culture conditions rather than discrete macrophage populations in vivo. Quantitative mechanical comparisons are valid only within the same cellular model and under comparable measurement conditions.

**Table 4:** Representative study-specific quantitative findings on biomechanical and related morphological changes in red blood cells, neutrophils, and macrophages.

Study, Ref.	Cell Type or Model	Method and Original Experimental Conditions	Quantitative Outcome
Besedina et al. (2022) [27]	Human RBCs from healthy donors (18 donors)	Microfluidics using channels approximately 2.5 × 8.0 μm in cross-section and 200 μm in length, and AFM in liquid; exposure to 0–1.5 mM tBuOOH for 4–5 h at 37 °C	At 1.5 mM tBuOOH, Young’s modulus increased from 66.8 to 131.9 kPa and probe adhesion from 30.4 to 48.5 pN. Slow-transiting cells accounted for 51.1% of the population, and channel occlusion occurred in approximately 76% of events
Kozlova et al. (2022) [43]	Human donor RBCs	AFM after exposure to Fe^2+^ at 1700 μM for 1 or 24 h	Young’s modulus increased from 11 ± 4 to 17 ± 5 kPa after 1 h and from 15 ± 5 to 23 ± 8 kPa after 24 h
Sergunova et al. (2022) [48]	Stored human RBCs	AFM in PBS after 15 min of hemin exposure, packed RBCs stored for 2–6 days served as controls	Young’s modulus increased from 10 ± 4 to 28.1 ± 7.5 kPa
Sergunova et al. (2026) [36]	Patient-derived RBCs: normothermic CPB (n = 7) and CPB combined with HCA at 28 °C (n = 7)	AFM before anesthesia and immediately after CPB, Young’s modulus was measured in PBS, and surface roughness was assessed on dried smears	Mean Young’s modulus increased 1.55-fold after normothermic CPB and 1.14-fold after CPB combined with HCA. Mean surface roughness increased 1.4- and 1.6-fold, respectively
Sherstyukova et al. (2025) [37]	Partially leukoreduced low-titer group O whole blood: control and pathogen-reduction groups	Riboflavin/UV pathogen reduction; storage at 4–6 °C for 7 days followed by freezing and thawing; AFM	Young’s modulus remained approximately 5 ± 2 kPa throughout storage and after thawing, without significant differences between groups (*p* > 0.05). On day 7, pathogen-reduced samples contained 63 ± 5% discocytes and 32 ± 6% echinocytes; hemolysis remained below 0.8%
Bashant et al. (2019) [8]	Human neutrophils in whole blood and isolated preparations	Real-time deformability cytometry after priming with fMLF, PAF, GM-CSF, or LPS; 20 × 20 μm channel and total flow rate of 0.12 μL s^−1^	Priming caused early cellular contraction and stiffening, followed by an approximately 18% increase in cell size and subsequent softening. By 45 min after fMLF stimulation, stiffness returned to values similar to those of unstimulated cells
Linderkamp et al. (1999) [58]	Neutrophils from children with newly diagnosed type 1 diabetes before insulin treatment and after 4–6 weeks of therapy; children treated for 5–8 years and healthy controls (n = 15 per group)	Micropipette aspiration using a 2.5 μm pipette and an aspiration pressure of 2 cm H_2_O; membrane–cytoplasm tongue length was measured after 60 s	Neutrophil tongue length was 5.8 ± 1.0 μm before insulin treatment, 6.9 ± 1.2 μm after 4–6 weeks of treatment, 7.7 ± 1.3 μm after 5–8 years of treatment, and 9.5 ± 1.4 μm in healthy children. Active neutrophils accounted for 9 ± 6% before treatment and 3 ± 2% in healthy children.
Inozemtsev et al. (2023) [10]	Primary human neutrophils stimulated with PMA or A23187	Widefield and confocal fluorescence microscopy, stimulation with 35 nM PMA or 2.5 μM A23187, observations from 30 s to 240 min	At 120 min, NETotic cells accounted for 14% after A23187 and 8% after PMA stimulation. NETosis reached 66% at 180 min with PMA and 48% at 240 min with A23187. Chromatin volume increased approximately 6–8-fold, reaching 1018.51 ± 491.93 μm^3^ after A23187 and 970.96 ± 333.59 μm^3^ after PMA stimulation. These values represent quantitative morphological rather than direct mechanical measurements
Neubert et al. (2018) [54]	Primary human neutrophils undergoing PMA-induced NETosis	Time-resolved fluorescence microscopy and AFM force measurements	Young’s modulus decreased from >1.5 to <0.3 kPa and membrane tension from >0.35 to <0.07 mN m^−1^. Chromatin swelling generated an internal pressure of approximately 100–200 Pa before plasma-membrane rupture
Petrova et al. (2026) [15]	Primary murine BMDMs: M0, LPS-induced M1-like, and IL-4/IL-10-induced M2-like states	AFM in PBS at 24 °C using a 2 μm spherical probe; n = 50 cells per group, siRNA-mediated Irf5 knockdown	Young’s modulus was 0.75 ± 0.55 kPa in M0, 0.80 ± 0.43 kPa in M1-like, and 1.54 ± 0.81 kPa in M2-like macrophages. Following Irf5 knockdown, Young’s modulus of M1-like macrophages increased to 1.36 ± 0.34 kPa
Wu et al. (2023) [16]	Live RAW 264.7 macrophages: M0, LPS-induced M1-like, and IL-4-induced M2-like states	AFM force–volume mapping at 37 °C	The mean full-indentation Young’s modulus of M0 cells was approximately 150 Pa. M1-like polarization produced a marked increase, whereas the increase in M2-like cells was smaller

Notes: Values are reproduced as reported in the cited studies and should not be interpreted as common reference ranges.

## Data Availability

No new data were created or analyzed in this study. Data sharing is not applicable to this article.
